# Bone Repair and Regenerative Biomaterials: Towards Recapitulating the Microenvironment

**DOI:** 10.3390/polym11091437

**Published:** 2019-09-02

**Authors:** Neda Aslankoohi, Dibakar Mondal, Amin S. Rizkalla, Kibret Mequanint

**Affiliations:** 1 School of Biomedical Engineering, The University of Western Ontario, London, ON N6A 5B9, Canada (N.A.) (A.S.R.); 2 Department of Chemical and Biochemical Engineering, The University of Western Ontario, London, ON N6A 5B9, Canada; 3 Schulich School of Medicine and Dentistry, The University of Western Ontario, London, ON N6A 5B9, Canada

**Keywords:** bone tissue engineering, biodegradable bioceramics, bioactive organic/inorganic hybrid biomaterials, sol-gel process, stem cells

## Abstract

Biomaterials and tissue engineering scaffolds play a central role to repair bone defects. Although ceramic derivatives have been historically used to repair bone, hybrid materials have emerged as viable alternatives. The rationale for hybrid bone biomaterials is to recapitulate the native bone composition to which these materials are intended to replace. In addition to the mechanical and dimensional stability, bone repair scaffolds are needed to provide suitable microenvironments for cells. Therefore, scaffolds serve more than a mere structural template suggesting a need for better and interactive biomaterials. In this review article, we aim to provide a summary of the current materials used in bone tissue engineering. Due to the ever-increasing scientific publications on this topic, this review cannot be exhaustive; however, we attempted to provide readers with the latest advance without being redundant. Furthermore, every attempt is made to ensure that seminal works and significant research findings are included, with minimal bias. After a concise review of crystalline calcium phosphates and non-crystalline bioactive glasses, the remaining sections of the manuscript are focused on organic-inorganic hybrid materials.

## 1. Introduction

As a major structural tissue of the human body, bone provides support and protection of various organs, regulates blood pH, produces bone marrow cells, and provides the microenvironments for multiple progenitor cells (hematopoietic and mesenchymal) [1,2,3,4,5]. Bone related disorders and subsequent bone loss are major burdens in terms of quality of life, health care costs, and economic impact. Bone injuries and defects can arise from a variety of causes, including bone diseases such as osteoporosis [6], osteoarthritis [7], osteogenesis imperfecta [8], osteomyelitis [9], etc. traumatic injury, orthopedic surgeries (i.e., total joint arthroplasty, spine arthrodesis, implant fixation, etc.) [10] and primary tumor resection [11]. 

Bone is a tissue known for its innate high regenerative capacity; however, in cases of critical-size bone defects, a tissue substitute or a biomaterial must be used to fill the gap or non-union. Although there is no clear definition for what constitutes a critical defect, it encompasses any defect that does not heal spontaneously. Anatomical location, the percentage of bone loss and the state of surrounding soft tissue are among the determining factors in this definition [12]. Consequently, treatment of critical bone defects necessitates surgical interventions. The current “gold standard” treatment of critical-size bone defects is autologous bone grafting in which, bone collected from the patient’s own body (typically from the pelvis or iliac crest) is utilized. However, the availability of autologous bone graft is limited and causes severe complications such as donor site morbidity, pain, paresthesia, prolonged hospitalization and rehabilitation, increased risk of deep infection, hematoma, inflammation, etc. [13]. Bone tissues collected from other humans (typically cadavers), also known as allografts can be alternative options; however, these carry risks of donor-to-recipient infection and disease transmission and host immune responses [13,14]. Another source of bone tissue can be non-human but bone xenografts are now widely considered to be unsuitable for transplantation due to the real and perceived risk of disease or virus transmission, infection, toxicity associated with sterilization, immunogenicity, and finally host rejection [15,16,17]. Consequently, synthetic bone grafts have emerged with several advantages such as elimination of disease transmission risk, availability of synthetic materials and reduced number of surgical procedures. Bone graft substitutes have evolved over the years, beginning from the first generation of inert materials developed over 40 years ago [18]. A common drawback of this first generation of bone grafts was the formation of fibrous tissue at the interface, which eventually isolates the graft from the surrounding tissue [19]. The desire for favorable interface led to the development of second-generation bioactive materials bone grafts in which bonding to the bone facilitates a positive interaction [20]. Bioactive glasses (BG), initially developed by the Hench laboratory, have been studied extensively. The Hench 45S5 BG (containing 45 wt.% SiO_2_, 24.5 wt.% CaO, 24.5 wt.% Na_2_O and 6 wt.% P_2_O_5_) has the ability to bind to the natural bone through formation of a hydroxyapatite layer on its surface [21]. Although other synthetic materials such as polymers and metals lack this bioactivity and inorganic materials are superior in this sense; however, their brittleness and difficulty to process in the form of tough 3D porous structure, restricted their application for bone regeneration [22]. Biocompatible and biodegradable polymers are easier to process but they are not without a limitation in terms of the mechanical properties to match with the bone tissue and the ability to integrate into the native tissue.

Apart from the type of materials, to enhance the potential of new bone formation, mimicking the microenvironment of the natural bone is essential and this is where bone tissue engineering has the potential to fill the gap. Bone tissue engineering aims at recruiting different elements to be able to mimic the bone microstructure and microenvironment towards the final goal of tissue regeneration and repair. For a tissue-engineered construct to be able to stimulate new bone formation, it should recapitulate the bone microenvironment as closely as possible. The bone microenvironment is a combination of biochemical gradients (proteins, growth factors), physical factors (porosity, stiffness, mechanical stimulation), and cellular niche (primary, progenitor, and stem cells).

When tissue engineering strategies are considered for bone repair, the scaffold is the primary element. The porous scaffold can be implanted at the defect site directly (in vivo bone tissue engineering) where the body serves as the source of cells and the bioreactor or can be seeded with the osteogenic cells and matured in a bioreactor by providing all required nutrients, oxygen, and bone inducing growth factors prior to implantation (in vitro bone tissue engineering) [23]. Clearly, scaffolds serve beyond a structural template and different designs and materials used for bone scaffold preparation.

## 2. Bone Tissue Engineering Scaffolds Are More than Mere Structural Templates

Seemingly, tissue engineering scaffolds including those designed for bone are viewed as structural templates with specific morphologies. However, they must mimic the natural extracellular matrix (ECM) which they are intended to replace mechanically, structurally, and biologically. Scaffolds act as a 3D support for cell adhesion, proliferation, and differentiation, and guide subsequent new bone formation. Therefore, an ideal scaffold should be osteoconductive thus that bone primary and progenitor cells can adhere and migrate onto the surface and eventually through the scaffold and begin to proliferate before laying down new matrix [24]. To allow cell migration as well as metabolic waste removal and angiogenesis, the scaffold should have a suitable pore size and interconnectivity [25]. The pore diameter of the scaffolds required to be larger than 100 µm for successful diffusion of the needed nutrients and oxygen for cell functionality [26]. Moreover, pore sizes in the range of 200 to 350 µm are found to be optimum for cell infiltration and bone tissue in-growth [27]. Furthermore, the scaffold should also be osteo-inductive thus that it can induce new bone formation by recruiting progenitor cells through biomolecular signaling. The intent of bone repair and regeneration is that the template scaffold should be biodegradable and replaced with new bone. This suggests the need for biodegradable materials to be selected and the degradation rate must be compatible with the rate of bone formation thus that newly formed bone can replace the scaffold. If the scaffold degradation rate is faster than new bone formation, some parts of the scaffold will be lost before ECM formation and immature bone grafts with large gaps will be formed. In addition, rapid degradation causes mechanical instability of the scaffolds. On the contrary, if the rate of degradation is slower than bone formation, newly formed ECM can cover up outer edges and a necrotic core can be developed due to the limitations of cell infiltration and nutrient exchanges [28,29]. The scaffold has to be mechanically competent to support bone formation. As a single material cannot provide all these requirements, bioactive composites, made of organic and inorganic bone-bioactive materials, could be good candidates for this application. Naturally, bone is an organic-inorganic composite material consisted of collagen and non-collagen proteins as the organic part and hydroxyapatite as the inorganic part [30]. Therefore, inspiring by nature has led to the development of organic-inorganic hybrids and composites. However, conventional composites consist of distinct phases, resulting in non-uniform physical, chemical, mechanical, and biological properties, making them unsuitable as bone biomaterials [22]. On the other hand, in the case of hybrid materials, the organic and inorganic components form an interpenetrating network at the nanoscale and behave as a single-phase material. Class I hybrids are characterized by non-covalent interactions, such as dipole-dipole interaction, hydrogen bonds, and van der Waal’s forces, between the components [31]. Class II hybrids are characterized by stronger interactions, such as covalent bonding between the organic and inorganic components [32]. By carefully choosing the organic and inorganic moieties and the synthetic approach, novel materials with tailored properties for a biological environment can be designed.

## 3. Bone Tissue Engineering Scaffolds Encompass the Chemistry of Inorganic and Organic Polymers

Development of suitable materials for bone tissue engineering scaffolds is the subject of ongoing discovery and innovation. Not surprisingly, the composite nature of the native bone tissue necessitated the exploration of both organic and inorganic polymers. Due to the multitude of approaches these two polymers can be combined, several materials have been proposed, characterized, and tested as potential biomaterials for fabricating bone tissue engineering scaffolds. However, as bioactivity and biodegradability are critical design parameters, the choice of materials is somewhat limited to degradable bioceramics and biopolymers. The following section will discuss some of the fundamental features of these biomaterials.

### 3.1. Biodegradable Inorganic Materials: Crystalline Calcium Phosphates and Non-crystalline Bioactive Glasses

#### 3.1.1. Crystalline Calcium Phosphates

Since non-degradable bioceramics form a non-adherent necrotic core isolated from surrounding bone, contemporary research efforts are devoted to biodegradable and bioactive bioceramics for tissue engineering applications [33]. Synthetic calcium phosphates (CaP) are osteoconductive, bioresorbable, and mimic the inorganic constituents of natural bone, which is a calcium phosphate in the form of carbonate apatite nanocrystals [34,35]. Based on composition, calcium phosphates for bone repair are classified as (i) calcium-deficient apatite, CDA (i.e., Ca/P molar ratio less than the stoichiometric value of 1.67 for pure hydroxyapatite), (ii) hydroxyapatite (HA), Ca_10_(PO_4_)_6_(OH)_2_, (iii) beta-tricalcium phosphate (β-TCP), Ca_3_(PO_4_)_2_, and (iv) biphasic calcium phosphate (BCP), an intimate mixture of HA and β-TCP of varying HA/β-TCP weight ratios. CaP powder can be prepared by a variety of wet chemical methods (such as precipitation, hydrolysis, hydrothermal, etc.) and solid-state reactions [36,37,38,39]. The synthesis of compact and dense CaP powder or scaffolds for bone regeneration often requires high-temperature sintering at 1000–1200 °C. Degradation of CaP in vitro or in vivo depends on their composition, physical shape, crystallinity, porosity, and preparation conditions [40,41,42]. Several in vitro and in vivo experiments have proven that the degradation or dissolution rate proceeds in the following decreasing order: Amorphous HA > α-TCP > β-TCP > crystalline HA. In the case of BCPs, degradation depends on the HA/β-TCP ratio, wherein the higher the ratio, the lower the degradation rate [43]. The rate of synthetic unsintered CaP degradation decreases in the order of amorphous calcium phosphate (ACP) > octacalcium phosphate (OCP) > CDA. 

Bioactivity of CaP bioceramics has been observed by direct attachment to the native bone on HA-coated biomaterials surface or as composites, while fibrous tissue encapsulates the uncoated surface [44,45,46,47]. Biomimetic carbonated apatite formation on CaP surfaces in simulated body fluid (SBF) as in vitro bioactivity was also evidenced by the uptake of calcium and phosphate ions from the solution [48,49]. CaPs allow osteoblast cells to attach, proliferate, and differentiate [50]. Differentiating osteoblast cells seeded on BCPs produce collagen (type 1), alkaline phosphatase, proteoglycans (decorin, lumican, biglycan), and matrix proteins (osteocalcin, osteopontin and bone sialoprotein) known to signify bone formation [51,52,53]. CaP coatings on bioinert materials for total joint arthroplasty have shown improved osseointegration at bone/implant interface resulting in superior implant stability [54]. Ectopic bone formation in vivo was also evidenced when CaP coated implants were inserted in non-osseous sites [55,56]. Despite their osteoconductive nature, the synthesis of compact and dense HA and TCP scaffolds for bone regeneration often requires high-temperature sintering and are poorly degradable in their highly crystalline form, while their amorphous counterparts are mechanically too brittle to be used for fabrication of highly porous scaffolds [34,57]. Sintered HA at high temperature exhibited high chemical stability in contact with tissue fluids, which leads to limited bioactivity and osteoconductive effect [58]. Instead, their amorphous counterparts are characterized by a high dissolution rate in vivo, which accelerates material desorption and incomplete tissue formation.

#### 3.1.2. Non-Crystalline Bioactive Glasses

Bioactive glasses (BGs) are a class of non-crystalline silicate glasses, which can stimulate bone-like mineral formation (hydroxy carbonate apatite, HCA) in the presence of physiological fluids. HCA is similar to the inorganic constituent of natural bone and it is believed that HCA layer interacts with bone ECM to bond with the native bone [59]. BG was first invented in 1969 and consisted of 46.1 mol % SiO_2_, 24.4 mol % Na_2_O, 26.9 mol % CaO and 2.6 mol % P_2_O_5_, later named as 45S5 and Bioglass^®^, which formed a strong bond with native bone during in vivo studies due to the HCA layer formation at bone-implant interfaces following the dissolution of glass materials [60]. Since then, several types of BGs have been developed by varying the compositions of constituents such as silicate-based, phosphate-based, and borate-based glasses [61,62].

Silicate based BGs are referred to those glasses in which (SiO_4_)^4−^ acts as the main 3D glass-forming networks. The SiO_2_ concentration in silicate-based glasses varied from 45–71 wt.%. Other components such as Na_2_O, K_2_O, MgO, CaO, P_2_O_5_, etc. used at various amounts as network modifiers [61,63,64,65]. Phosphate-based glasses have a [PO_4_]^3−^ structural unit as the main network former and CaO and Na_2_O as modifiers. Many studies have shown their potential for tissue engineering applications [66,67]. Phosphate-based glasses have a chemical affinity towards bone due to the similarity to inorganic phases of bone. These type of BGs have a high dissolution rate in aqueous media due to the ease of P–O–P bond hydration [68,69]. As their dissolution rate is strongly composition-dependent and can be tailored by adding appropriate metal oxides, such as TiO_2_, CuO, NiO, MnO, Fe_2_O_3_, etc. to the glass composition, they have been widely investigated as controlled release vehicles for antibacterial ions for tissue engineering [70,71].

Over the past 15 years, investigations have shown that borate-based BGs are bioactive and due to their faster rate of dissolution, they enhanced HCA mineral formation in vitro, when compared to some silicate-based glasses [72,73,74,75,76,77,78]. Boron containing BGs inhibit the formation of slightly more stable SiO_2_-rich layer at an early stage of dissolution in the physiological medium, which results in a faster rate of dissolution and rapid HCA formation [61,62,72,75,76]. For bone tissue engineering applications, tailoring the rate of degradation of biomaterial scaffold is vital. Modification of BG composition allows to control their degradation rate in vitro and enhance the regeneration of bone. For instance, by partially replacing the SiO_2_ in silicate-based BGs with B_2_O_3_, the degradation rate can be varied over a wide range [74,75]. This way it is possible to match the degradation rate of borate-based BGs with the rate of de novo bone ECM formation. On the other hand, boron as a trace element is required for maintaining bone health [79]. Borate-based BGs support cell proliferation along with differentiation in vitro, whereas in vivo studies reported that Boron enhances tissue infiltration [80,81,82]. These BGs have also shown to serve as a substrate for drug release in the treatment of bone infection [83,84,85]. Despite their excellent bioactivity, some investigations indicated that certain compositions of borate-based BGs exhibited cytotoxicity under static in vitro culture conditions, whereas no considerable toxicity was detected under dynamic culture [75,85]. A scaffold made of borate-based BG by replacing all the SiO_2_ with B_2_O_3_ were found to be toxic to murine MLO-A5 osteogenic cells in vitro [82]. However, the same scaffolds did not show any toxicity to cells in vivo and supported new tissue infiltration when implanted subcutaneously in rats [82,86]. The concentration of boron in culture media entirely depends on the initial composition of B_2_O_3_ in glass and it can be regulated by optimization. The adhesion and proliferation of bone marrow stromal cells were enhanced due to the conversion of the glass to HCA when borate ions were released into the culture media from BG containing low boron initially [62]. Osteoconductivity and bioactivity of borate-based BGs make them promising candidates for bone tissue engineering applications.

Initially, BGs were synthesized through melt-quenching techniques by mixing and melting ceramic powders such as SiO_2_, P_2_O_5_, CaO, Na_2_O, etc. above 1300 °C, followed by quenching in graphite mold or in cold water [60]. Since then, BGs were prepared this way until the early 1990s when sol-gel synthesis of BGs was introduced [87,88]. The sol-gel synthesis route allows chemistry-based room-temperature synthesis of BGs where colloidal suspensions (sol) of glass precursors undergo a series of hydrolysis and polycondensation reactions to form a gel. The gel is an inorganic network of covalently bonded glass components, which can then be dried to become a glass monolith. The glass precursors are in the form of metal alkoxides and have the generic structure M–(OR)x, where a central metallic ion (M) is bound to functional organic groups, mainly alkyl (–OR). Metal alkoxides, such as tetraethyl orthosilicate (TEOS) or tetramethyl orthosilicate (TMOS) are often used as SiO_2_ precursors and triethyl phosphate (TEP) as P_2_O_5_ precursor due to their abilities to readily react with water. The acid or base catalyzed hydrolysis reaction results in the replacement of alkoxy side chains with hydroxyl groups. Hydrolysis occurs through a nucleophilic attack on the core atom (e.g., Si) by the oxygen atom in the water molecule [88].

Hydrolysis: M–(OR)_4_ + 4(H_2_O) → HO–M(OR)_3_ + R–OH (1)

where, –OR represents an alkoxy functional group, e.g., C_2_H_5_O–.

Subsequent polycondensation reactions result in covalently bonded inorganic glass network formation.

Condensation: (OR)_3_M–OR + HO–M(OR)_3_ → (OR)_3_M–O–M(OR)_3_ + R–OH (2)

and/or,

HO–M(OR)_3_ + HO–M(OR)_3_ → (OR)_3_M–O–M(OR)_3_ + H_2_O (3)

The nature of final inorganic glass networks depends on pH, acid or base catalysts, solvent-reactant ratios and precursor molecules [89]. Major differences between melt and sol-gel derived BGs are that sol-gel glasses tend to have higher purity, homogenous microstructure, and nanoporosity, whereas melt-quenched BGs have heterogeneous phase distribution and dense microstructure [90]. The increased nanoporosity and surface area allow improved cellular response and bioresorbability of the BGs [91]. In addition, due to the room-temperature synthesis, there is no need to include Na_2_O to decrease the melting temperature and also there are a large number of silanol groups on the external surface of the BG network. These, in turn, enable the organic modification of the glass, which is essential to prepare osteo-inductive scaffolds, by grafting the biomaterials with different active agents, such as certain peptides, proteins, and growth factors.

Major disadvantages of BGs are their stiffness and requirement of high temperature to process into desired shapes rather than just powders. In addition, it is challenging to prevent cracks during the drying stage of sol-gel derived BGs monoliths. Cracking occurs due to the large shrinkage during drying, and evaporation of the liquid by-products of the condensation reaction. When liquid from pore vicinity is removed from the gels, it must travel from inside of the gel to the surface via the interconnected pore network. This causes capillary stresses within the pore network and, therefore, cracking occurs [59]. For powders, coatings or fibers, the capillary stresses are nominal, as the evaporation path is short and the stresses can be accommodated by the material. For monolithic objects such as scaffolds, the path from the center of the monolith to the surface is long and tortuous, and the drying stresses can introduce catastrophic fracture with increasing pore size and obtaining pores with a narrow distribution. These disadvantages can be overcome by preparing composites of BGs with degradable biopolymers.

### 3.2. Biocompatible and Degradable Organic Polymers

Both natural and synthetic biocompatible organic polymers have been widely investigated for bone tissue engineering applications [92,93,94,95]. The main forms of natural biodegradable polymers are proteins such as collagen, gelatin, albumin, etc. and polysaccharides such as cellulose, hyaluronate, chitin, alginate, etc. Synthetic polymers such as polyglycolic acid (PGA), poly(L-lactic acid) (PLA), poly(lactic-co-glycolide) (PLGA), polycaprolactone(PCL), poly(propylene fumarate) (PPF), polyhydroxyalkanoates (PHA), polyanhydrides, polyorthoesters, poly(vinylpyrrolidone) (PVP), polyphosphazene, etc. are prominent candidates for bone tissue engineering applications. Although naturally derived polymers have shown better cell-material interactions, major drawbacks such as their availability in large amounts, difficulties in processing, and purification have encouraged researchers to explore the use of synthetic polymers [93,96]. Synthetic polymers are advantageous compared to natural polymers since their properties such as porosity, mechanical properties, and degradation rate can be tailored for specific applications. Synthetic polymers are often more available than naturally derived polymers, they can be produced in large reproducible quantities without batch to batch variability seen in natural polymers, and have a long shelf time. Many commercially available synthetic polymers exhibit physicochemical and mechanical properties comparable to those of biological tissues. They exhibit, in general, predictable and reproducible mechanical and physical properties such as tensile strength, elastic modulus, and degradation rate and can be processed into different shapes. 

Although poly(α-hydroxy esters) such as PGA, PLA, PCL, and their copolymers are degraded by hydrolysis and can be metabolized and excreted, their biocompatibility sometimes is challenged by the acidic degradation products [97]. Moreover, polymeric biomaterials, in general, have limited strength and mechanical stability when made with large volume fractions of macro-porosity, which is a desired design consideration for tissue regenerative materials. These polymers undergo a bulk erosion process such that it can cause scaffolds to fail prematurely [98]. In addition, they are not osteoconductive and do not adequately promote bone cells to adhere, grow, and proliferate.

The scaffolds applied for bone tissue engineering should be osteoconductive and osteoinductive, having suitable porous 3D microstructure to allow cell infiltration, tissue growth, and metabolic waste removal. Their rate of degradation should also be compatible with the rate of new bone formation thus that the newly formed bone can replace the scaffold. One of the challenges associated with developing scaffolds for bone tissue engineering is that no single material meets the above-mentioned properties. Composites of materials with desired properties consisting of organic and inorganic components have been proposed to be a solution to this problem.

### 3.3. Composite Bone Biomaterials from Inorganic and Organic Polymers

**Figure 1:**
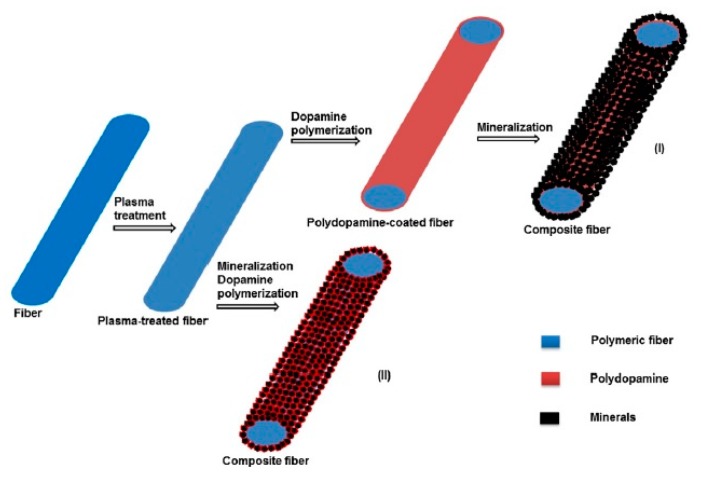
Schematic illustration of the formation of composite fibers. Method I: Plasma-treated electrospun polycaprolactone (PCL) fibers were coated with polydopamine prior to mineralization in 10xSBF. Method II: Plasma-treated electrospun PCL fibers were mineralized in the 10xSBF solution containing dopamine. Reproduced with permission from reference [107].

Organic-inorganic (O/I) composites prepared from BGs and biodegradable polymers gained much attention due to the advantages of combining their properties, and the possibility to obtain required bioactivity, degradation behavior, and mechanical properties for bone tissue engineering scaffold. Toughness and processability of polymers can be added with excellent bioactivity of BGs by preparing composite materials [33]. Generally, these composites were prepared by using the polymers as matrix and BG powders as filler. Composite scaffolds prepared with BGs and polymers such as PCL, PLA, PGA, PLGA, etc. have shown improved mechanical properties compared with pure BGs or pure polymers [99,100,101,102,103]. A thin coating of PDLLA [104] or PHB [105] have also been applied to BG foam scaffolds to improve the fracture resistance. However, the efficacy of enhancing bioactivity or improving mechanical properties through introducing BG in polymer-coated scaffolds is questionable since coated polymer would cover BG surfaces until the polymer is fully degraded, and when it degrades only the brittle BG scaffold will be left [59]. This is also true for conventional composites where micro- or nano-sized BG particles are embedded in a polymer matrix, and bone cells come into contact with the polymer mostly. Thus, the resultant bioactivity and cell-material interactions decrease for such composite biomaterials [59]. This is the reason that some studies tried to deposit minerals on the surface of polymeric scaffolds, however, providing interaction in the interface is challenging and different secondary processing such as plasma treatment or surface modification such as heparin, polydopamine, etc. have been applied to provide better attachment of the deposited layer (Figure 1) [106,107,108,109,110,111]. Preparing composites of BGs and bioresorbable polymers also modifies the degradation behavior of polymers. Polymers acidic degradation by-products may cause toxicity to cells, while BG degrades with releasing cations, which can buffer the acidic by-products and maintain neutral pH at O/I interfaces [101,112]. BGs are hydrophilic and incorporation of BGs in hydrophobic polymer matrices also alters the surface and bulk properties of O/I composite scaffolds by increasing the hydrophilicity and water absorption hence modifies the degradation kinetics [113]. However, it is difficult to match the degradation rates of the two components in O/I composites [114]. In an ideal condition, both the polymer and BG phases should degrade in consort and at a suitable rate thus that the scaffolds can gradually be replaced by the newly formed tissue, as well as maintain their mechanical integrity to support and guide bone regeneration. In conventional O/I composites, different phases degrade at different rates, which cause non-uniform dissolution and mechanical instability of the scaffolds. An alternative way to overcome these non-uniform properties is to produce O/I nanocomposites in which inorganic nanoparticles or nanofibers are blended with a polymer matrix [115,116,117]. 

Organic/Inorganic nanocomposites prepared with nano-sized BG filler provide a larger surface area compared to conventional composites (prepared with micro-sized BG particles). This increased surface area of BG positively affects the cell-material interactions. Nanoparticles of bioceramics enhanced protein adsorption and osteoblast adhesion when compared with their micro-sized counterparts [118]. To yield O/I nanocomposites with improved bioactivity, cell-material interactions, and mechanical properties, the nanoparticle size is an important parameter. It was observed in a detailed study on porous 3D PLLA/BG nanocomposite scaffolds that addition of BG nanoparticles up to 20 wt.% did not change their morphology but enhanced their bioactivity [119,120,121]. With increasing the amount of BG from 0 to 30 wt.%, the compressive modulus of the nanocomposite scaffolds increased from 5.5 to 8.0 MPa. Incorporation of BG nanoparticles with the PLLA matrix also assisted the increase in the equilibrium water uptake of the nanocomposite scaffolds and affected the rate of degradation. BG nanofibers were also used to fabricate nanocomposite scaffolds. Sol-gel derived electrospun BG nanofibers [122] were combined with several biodegradable polymers. These resulted in good bioactivity and HCA deposition on their surfaces when exposed to SBF [123,124,125,126]. In addition, these nanocomposites induced the osteoblast-like cells attachment, spreading and proliferation in vitro. In general, O/I nanocomposites prepared with BG nanoparticles or nanofibers demonstrated better mechanical properties and cell-material interactions compared to the conventional micro-composites due to their higher surface area to volume ratio. 

Nanocomposites of nanohydroxyapatite particles in a polymeric matrix is another highly investigated O/I nanocomposite [127,128,129,130]. However, the challenge to synchronize the degradation rates of different phases in O/I nanocomposites still exists. The mismatch between the degradation rates of the different phases may cause failure in the long-term in vitro operations. Moreover, nanoparticles are prone to particle agglomeration and homogenous distribution of inorganic BG particles in a polymer matrix is difficult to achieve if there are no physical or chemical interactions between organic and inorganic phases [59]. A viable approach to achieve homogenous dispersion can be adding the polymer in the sol during the sol-gel synthesis of BG. However, without having bonding sites in the polymer chain, this causes organic-inorganic phase separations and micro- or nano-sized phases in the final composites [131]. Therefore, in view of the above discussion, it is obvious that the molecular level interactions are required among the organic-inorganic phases to fabricate suitable biomaterial scaffolds for bone tissue engineering applications.

### 3.4. Hybrid Bone Biomaterials from Inorganic and Organic Polymers

**Figure 2:**
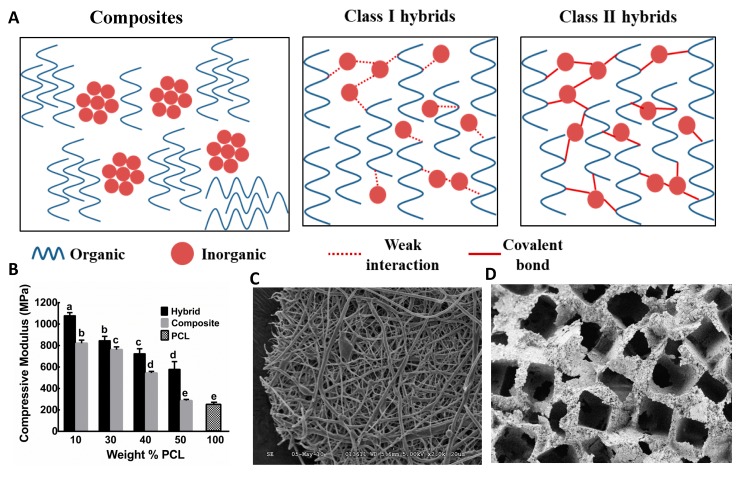
(**A**) Interaction between phases in composites and organic-inorganic (O/I) hybrid materials (**B**) compressive modulus of class II PCL–BPSG (borophosphosilicate glass) hybrid compared to composite of PCL and BPSG and PCL alone [146] (**C**) SEM image of electrospun class I PCL-tertiary bioactive glass (unpublished data from Mequanint Lab) (**D**) class II PCL–BPSG hybrid scaffold fabricated by solvent-free casting and particulate leaching [78]. Reproduced with permission from the cited references.

Generally, O/I hybrid biomaterials are defined as organic and inorganic biomaterials blended on a molecular scale and the phases are indistinctive in and above nanoscale [32]. Interpenetrating networks of organic and inorganic biomaterials interact below nanoscale and form O/I hybrid biomaterials. Hybrids are different from nanocomposites, in which the phases in the hybrids are indistinguishable on a nanoscale [132]. O/I hybrid biomaterials exhibit homogeneous dispersions of organic and inorganic components as building blocks or interpenetrating networks. Due to their high degree of organization on a molecular scale, hybrid biomaterials not only display intrinsic physical properties of the constituents organic and inorganic biomaterials but also new properties as synergistic effects [133]. As both organic and inorganic components need to be mixed on a molecular level, a low-temperature synthesis route such as the sol-gel process is required to prepare these biomaterials. The intimate molecular level interactions between phases promote the O/I hybrid material to act as a single-phase material with advantages of tailorable mechanical, chemical, and physical properties [134,135]. Since the chemical nature of organic and inorganic moieties are different, without having reactive sites in both components, phase separation can occur during the synthesis. Therefore, it is required to choose an appropriate polymer or functionalize the polymer prior to synthesizing hybrid biomaterials. Based on the nature of interactions, hybrid materials are categorized into two classes (Figure 2A). Class I hybrids have weak molecular interactions between the organic and inorganic phases, such as van der Waal’s, hydrogen bonding, or weak electrostatic interactions. Class II hybrids have strong chemical interactions such as covalent bonding between the components [32]. The following sections will discuss these two class of hybrid biomaterials and their applications as scaffold materials in bone tissue engineering.

#### 3.4.1. O/I Class I Hybrid Biomaterials

Bioactive glass-containing class I hybrid biomaterials are prepared through the sol-gel synthesis of inorganic BG in presence of polymer. During the formation of BG networks (Si–O–Si) through hydrolysis and polycondensation, organic polymers are entrapped within the inorganic glass network. The efficacy of class I hybrid formation entirely depends on the polymer interaction with the silanol (Si–OH) groups in the glass network. Reaction conditions and parameters are optimized carefully thus that the organic phase cannot be separated or precipitated during sol to gel and gel to dry monolith conversions, and hence optically transparent class I hybrid biomaterials can be obtained. 

Incorporation of polyvinyl alcohol (PVA) into the inorganic BG networks during sol-gel synthesis produced bioactive and crack-free O/I class I hybrid monoliths [136]. However, excess PVA content resulted in rapid disintegration when exposed to a buffer solution. Similar studies have shown that up to 30 wt.% PVA could be incorporated in PVA/BG hybrid [137,138]. The application of PVA based hybrid scaffolds in bone tissue engineering is limited due to the non-biodegradability of PVA. Class I gelatin/BG hybrids have been obtained through interaction between the silanol groups and the amino groups in the gelatin and scaffolds of these hybrids have been fabricated by thermally induced phase separation [139]. Polyethylene glycol (PEG) has also been used as the organic component of hybrid. The water solubility of PEG makes it a convenient choice for incorporation in the sol-gel process [140]. Another major concern of class I hybrid synthesis is the polymer needs to be soluble in the sol during the sol-gel process thus that it does not precipitate in liberated ethanol or water during the process.

Allo et al. prepared PCL/BG class I O/I hybrid monoliths and their nanofiber mesh scaffolds through the sol-gel process using methyl ethyl ketone (MEK) as a solvent for PCL (80 kDa) to avoid phase separation during the synthesis (Figure 2C) [31]. Detailed analyses have shown that class I hybrids were prepared with up to 60 wt.% of PCL through hydrogen bonding among carbonyl groups of the polymer backbone and silanol groups in BG. These hybrids have shown HCA deposition on 2D monolithic surfaces while incubated in SBF and favored excellent MC3T3-E1 preosteoblastic cell attachment and proliferation [141]. While prepared as 3D electrospun fibrous scaffolds, these hybrids exhibited good mechanical properties, and early expressions of transcription-level collagen type I (Col I), alkaline phosphatase (ALP), osteopontin (OPN), bone sialoprotein (BSP) and osteocalcin (OCN) genes [142]. These hybrid biomaterials have great potential for bone regeneration in vitro. However, the degradation study for this hybrid has not been investigated. Another drawback was that the electrospun fibrous scaffolds tended to have small pore sizes that may affect cell infiltration and scaffold remodeling. In addition to MEK, hexafluoro-2-propanol (HFP) has also been used as a water-miscible solvent for synthesis of PLLA/silica hybrid [143]. Electrospun fibers of PCL/silica hybrid showed nano-sized microstructures under TEM, which were uniformly self-organized throughout the hybrid fibers [144]. The same approach of using water-miscible solvents for the organic phase has been used for other systems such as polyhydroxybutyrate/poly(ε-caprolactone)/58S hybrids [145].

#### 3.4.2. O/I Class II Hybrid Biomaterials

Although hydrogen bonding or other weak interactions between the organic-inorganic moieties provide improved properties to the hybrid biomaterials compared to conventional composites, initial mechanical competency, predictable degradation behavior, and uniform bioactivity can be achieved through strong chemical bonding between the organic and inorganic phases (class II hybrids) [59]. Different strategies for the synthesis of class II hybrid biomaterials can be categorized into three main approaches: i) Utilization of a coupling agent that can bond with both organic and inorganic phases, ii) utilization of an organic polymer containing trialkoxysilane (–Si(OR)_3_) functional group(s), and iii) in situ polymerization of organic and inorganic phases from their precursor monomers. 

Covalent crosslinking between a degradable polymer and inorganic network can be obtained by using coupling agents. In this approach, the polymer should be functionalized with alkoxysilane functional groups prior to the sol-gel process. Coupling agents such as (3-glycidoxypropyl) trimethoxysilane (GPTMS), (3-isocyanatopropyl) triethoxysilane (ICPTES), (3-aminopropyl) triethoxysilane (APTES), etc., have been used to functionalize the polymers. Chitosan/SiO_2_ class II hybrids have been prepared using GPTMS as a coupling agent [147,148,149,150]. Epoxy groups from one end of GPTMS chemically bonded with -NH_2_ groups of chitosan chain and trimethoxysilane groups from the other end went through hydrolysis and polycondensation reactions with TEOS to form an organic-inorganic matrix. These hybrids have displayed tailorable hydrophilicity and controlled dissolution behavior as well as excellent cell-biomaterial interactions. Enhanced proliferation of osteoblastic cells on the surface of the hybrid, compared to chitosan alone, was attributed to the presence of silanol and siloxane bond as well as release of Si ions [151]. Scaffolds with different macroscopic geometries (sheet, disks, beads, etc.) have been fabricated from this hybrid by freeze drying technique [152]. Electrospinning has been utilized for fabrication of nanofibrous scaffolds of a hybrid of chitosan/polyethylene oxide and silica [153]. Hybrids of chitosan/gelatin and silica were also prepared through covalent incorporation of amine-containing siloxane through Schiff base formation into gelatin and chitosan [154].

Polycaprolactone diol (a low molecular weight PCL end-capped with two –OH) was coupled with ICPTES prior to synthesizing class II PCL/SiO_2_ hybrid through the reaction of –N=C=O groups from ICPTES with –OH [155,156]. These studies have shown that the coupling of PCL improved its solubility in the sol and the resultant hybrid had excellent mechanical properties. The lower molecular weight of the polymer assisted a uniform HCA layer deposition due to the faster rate of dissolution of PCL and hence higher exposed surface area of silica, which might have acted as nucleation sites. The bioactivity and mechanical properties of these PCL/SiO_2_ hybrids were dependent on the PCL content [155]. HCA deposition decreased with increment of PCL content, but toughness was increased. Other coupling agents such as 3-isocyanatopropyl triethoxysilane (IPTS) have also been used for synthesis of PCL/silica hybrids [157,158]. Poly(lactic-co-glycolic) acid/silica hybrids have also been synthesized by using IPTS as the coupling agent. The PLGA copolymer composition was used as a method for tailoring the degradation rate and subsequently investigating the effect of pH on the apatite-forming ability of the hybrid. It was shown that 50PLA50PGA samples with higher degradation rate did not show apatite-forming ability within 28 days comparable to PLGA with the composition of 90PLA10PGA, which showed apatite-forming ability within three days of incubation. This observation was attributed to the higher dissolution of apatite at pHs below 5.6 [159]. 

Multi-armed oligomers composed of an ethoxylated alcohol core and hydrolytically degradable oligo(d,l-lactide) units were used as the organic component of a hybrid. Different degrees of ethoxylation and varying length and content of oligoester units and varying content of organic component were used as tools to control biodegradation and mechanical properties of the hybrid [160]. The 2- to 4-armed organic crosslinkers with small organic core molecules functionalized with ICPTES were synthesized to crosslink a pure silica sol. Scaffolds fabricated by indirect rapid prototyping from this hybrid material were used for assessment of the effect of crosslinkers’ size and content on the mechanical properties [161]. 

**Figure 3:**
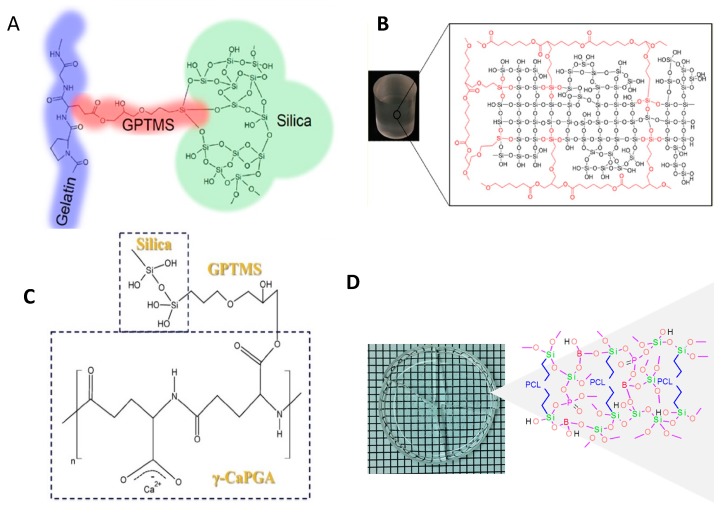
(**A**) Chemical structure of the linkage of organic and inorganic components by (3-glycidoxypropyl) trimethoxysilane (GPTMS) in class II gelatin/silica hybrids [162] (**B**) chemical structure of hybrids of silica and copolymer of caprolactone and glycidoxypropyl trimethoxysilane [163] (**C**) schematic illustration of the expected chemical structure of the calcium-containing silica/γ-PGA hybrid [164] (**D**) digital photo and chemical structure of class II PCL-borophosphosilicate hybrids [78]. Reproduced with permission from the cited references.

Gelatin/SiO_2_ and gelatin/BG class II hybrids were also prepared using GPTMS as a coupling agent (Figure 3A) [162,165,166]. The dissolution of gelatin and silica decreased with the increase in the GPTMS amount due to the covalent crosslinking between organic-inorganic phases. The compressive properties were also increased with the increase in the covalent coupling. It has been observed that a two-fold increase in the GPTMS amount, resulted in a 360% increase in the stiffness values [165]. Increasing the molecular weight of gelatin is another way for enhancing the stiffness of the material [162]. Electrospun gelatin/BG hybrids have been also obtained by using GPTMS as a crosslinker. However, the incorporation of calcium within the network was unproven [167] and seems to be unlikely as well, since it is known that in the case of using calcium nitrate as the calcium source, thermal treatment above 400 °C is needed for calcium to be incorporated in the glass network [168]. However, class II hybrid of gelatin and SiO_2_–CaO BG have been synthesized using calcium ethoxide as a calcium source [169]. Class I and II of silica-gelatin hybrids have been processed via cryogenic solution blow spinning to produce fibrous structures in a rapid and scalable manner. Reversible conformation changes of gelatin from random coil to triple helix due to temperature change has been exploited for tailoring the viscosity of the hybrid for fiber spinning [170]. 3D robotic deposition has also been used for scaffold fabrication form gelatin/BG hybrids. In this case, to be able to have sufficient time for processing the hybrid, it is crosslinked using EDC-NHS chemistry after the scaffold fabrication process [171]. A major concern for using biopolymers such as gelatin is that the available functional groups are unknown and thus the amount of covalent crosslinking during the hybrid synthesis cannot be predicted. Another class II hybrid was prepared using poly(γ-glutamic acid) (γ-PGA) functionalized with GPTMS and SiO_2_ as the inorganic component [172]. By using GPTMS and controlling the degree of covalent coupling, the mechanical properties and dissolution rate of the hybrid can be tailored [173]. γ-PGA is an enzymatically degradable polymer based on amino acid glutamic acid and in the salt form is water soluble and, therefore, can be easily used in the sol-gel process. Electrospun scaffolds have also been fabricated from this hybrid [174,175]. Calcium salt of γ-PGA has also been used to incorporate calcium in the hybrid (Figure 3C) [164]. The calcium methoxyethoxide (CME) has also been used as a calcium alkoxide in the synthesis of γ-PGA hybrids, in which dilution of CME with DMSO and addition of this solution slowly at the last step of synthesis were attempted to control the gelation [176]. Low molecular weight PEG was used for synthesis of PEG/SiO_2_–CaO hybrids using CME as the calcium source. PEG ends were first modified with epoxy ethane, then ammoniapropyltriethoxysilane was added to react with epoxy ethane to form PEG end capped siloxane (PEGM). The compressive strength and Young’s modulus of the hybrid can be enhanced by increasing the molecular weight of the initial PEG chains [177]. Bis(3-aminopropyl) polyethylene glycol has also been used as a water-soluble organic component to synthesize class II hybrid with silica as the inorganic component and GPTMS as coupling agent [178]. 

There are numerous examples of the use of GPTMS as the silane coupling agent in the synthesis of class II hybrids based on the notion that the epoxy ring of GPTMS will be opened by water or carboxyl or amine groups and a bond between the GPTMS and the organic polymer will be formed. However, it should be noted that there will be simultaneous competitive reactions such as the hydrolysis and condensation of GPTMS itself. Therefore, the reaction conditions should be controlled carefully [178]. NMR and mass spectrometric analyses were used to investigate the extent of competing reactions that occur as a function of the pH value. It was concluded that for functionalization of the polymers with GPTMS, a slightly acidic condition is needed. However, in higher acidity, hydrolysis of the epoxide ring is the prevalent reaction. On the other hand, in basic conditions, the epoxide ring opening does not occur [179]. In the case of hybrid of chitosan and silica, it was shown that both reactions of GPTMS with water and with chitosan, are acid catalyzed and the relative amounts of product and side-product do not depend on pH for two tested pHs of 2 and 4 [147]. However, the pH does affect the condensation rate of the silica network and subsequently the dissolution rate of silica and the mechanical properties of the hybrid [147]. And regardless of the GPTMS crosslinker content, the crosslinking density does not exceed around 80% [149]. In the case of alginate/silica hybrids it was also observed that the amount of GPTMS coupling to the alginate was low, and a considerable portion of GPTMS formed diols or a separate network [180]. GPTMS has also been used as a co-monomer and crosslinker for the synthesis of PCL/silica hybrids. Unlike using GPTMS only as a coupling agent between PCL diol and silica network, copolymerization of PCL and GPTMS by ring opening polymerization results in control over the ratio of silane containing groups (from GPTMS) and caprolactone (CL) repeating units along the polymer chain (Figure 3B) [163]. 

**Figure 4:**
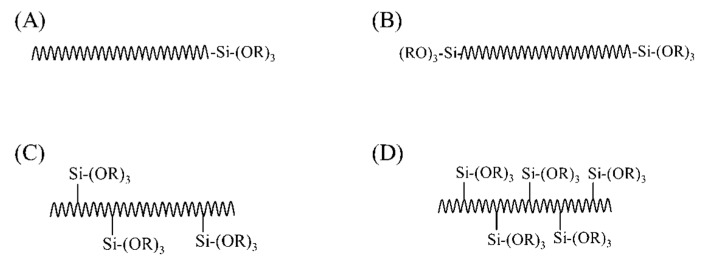
Various types of functionalization of polymer with trialkoxysilane (**A**) monofunctionalization (**B**) difunctionalization; pendant functionalization with side chains (**C**) random copolymerization (**D**) block copolymerization.

A disadvantage of using coupling agents in the synthesis of class II hybrids is that the functionalized polymers provide a limited number of functional groups in the polymer backbone. Therefore, polymers with high molecular weight would have very poor interaction with inorganic phases and this may cause phase separation over a certain amount of organic moiety [181]. Polymers with trialkoxysilane functional groups as side groups or pendant in the polymer backbone can be a better choice to synthesize class II hybrids through the sol-gel process due to the predictability of degree of crosslinking regardless of the molecular weight (Figure 4). For example, polydimethoxysilane (PDMS) contains functional silane side groups and has been used to prepare class II hybrids by hydrolysis and co-condensation with TEOS [182,183,184]. The in vitro bioactivity of these hybrids was dependent on incorporated Ca^2+^ in O/I networks, evaluated after incubation in SBF [185,186]. These studies have shown that bioactivity of these hybrids increased with higher inorganic content and, therefore, the mechanical properties enhanced. However, due to non-degradability, PDMS is not a viable choice for bone tissue engineering. 

Functionalization of the polymer with trialkoxysilane as side groups in the polymer backbone can be obtained easily by copolymerization of the monomer with an alkoxysilane monomer. The copolymer of polystyrene [187], poly(2-hydroxyethylmethacrylate) [188,189,190,191,192], acrylonitrile butadiene styrene [193], poly(methyl methacrylate) [194,195,196,197,198,199] were prepared with several trialkoxysilane (–Si(OR)_3_) monomers prior to introducing them into the sol-gel process. However, these polymers that were used to make the hybrids were not biodegradable or water-soluble, which restricted their application for bone regeneration. Introducing degradable segments to the polymer in a way that after cleavage, the products with molecular weights below the kidney filtration threshold are produced, is one of the proposed ways for the synthesis of degradable hybrids based on poly(methyl methacrylate) copolymers [200]. Moreover, these materials mostly contained SiO_2_ as the only inorganic component and, therefore, they were not sufficiently bioactive to induce osteogenesis [59].

#### 3.4.3. Challenges Associated with the Synthesis of Class II Hybrids

O/I class II hybrids are most sought-after biomaterial system for assuring uniform physical, mechanical, and biochemical properties at the molecular level. Covalent bonding between the organic and inorganic components is the key feature of the successful preparation of these novel materials. However, there are major chemical challenges in the synthesis of the O/I hybrid materials. These challenges are described below.

##### Synthesis Route

(i) Aqueous sol-gel process

Polymers having trialkoxysilane functional groups (–Si(OR)_3_) and BG precursors (TEOS, TEP, etc.) are hydrolyzed and polycondensed congruently in the standard sol-gel process to form O/I class II hybrids. Since the carbon-silicon bond is inert, hydrolysis of the functionalized polymer can easily help bond the inorganic network through Si–O–Si linkage through the polycondensation reaction. The room-temperature sol-gel process also prevents any sort of thermal degradation of organic contents [201,202]. As mentioned earlier, in the last decade, several O/I class I and II hybrid biomaterials have been prepared through the sol-gel process. These hybrids were prepared by carrying out standard aqueous sol-gel process, in which hydrolysis and polycondensation of trialkoxysilane (from polymer chains) and TEOS (to form inorganic network) occurred in the presence of water as reactant and solvent. This aqueous sol-gel synthesis limited the choice of water-soluble and/or hydrophilic biocompatible polymers. However, many synthetic biocompatible and degradable polymers are water-insoluble and have great potential as organic component of hybrid biomaterial to apply for bone regeneration in vitro. Water-miscible organic solvents can be used as co-solvents during the sol-gel synthesis to prevent the polymer from being phase-separated or precipitated. However, optimization of the volume ratio of solvents and removal of the co-solvent afterward are further challenges. Allo et al. prepared PCL/BG class I hybrid and Rhee et al. prepared PCL/SiO_2_ class II hybrid through aqueous sol-gel process using MEK and THF, respectively, as co-solvents to dissolve PCL [31,155]. Both solvents are cytotoxic and should be completely removed from the synthesized hybrids before seeding cells. Moreover, the process is not versatile due to PCL phase separation when the amount is increased above 60%.

(ii) Non- aqueous sol-gel process

The above limitations associated with the aqueous sol-gel synthesis of O/I hybrids may be addressed by non-aqueous (or non-hydrolytic) sol-gel process [203,204,205]. In this process, the organic precursors transform into sol by reacting them with carboxylic acid and the process takes place in an organic solvent. The carboxylic acid (e.g., formic acid, acetic acid, etc.) also may serve as the solvent. The non-aqueous sol-gel process starts with carboxylation of TEOS and trialkoxysilane. The reactions occur as follows:

Carboxylation: R′COOH + Si(OR)_4_ → (OR)_3_Si–OOCR′ + R–OH (4)

R′COOH + (OR)_3_Si–OH → (OR)_3_Si–OOCR′ + H_2_O (5)

Esterification: (OR)_3_Si–OOCR′ + R-OH → (OR)_3_Si–OH + R′COO–R (6)

R–OH + R′COOH → R′COO–R + H_2_O (7)

Condensation: (OR)_3_Si–OH + HO–Si(OR)_3_ → (OR)_3_Si–O–Si(OR)_3_ (8)

2Si(OH)_4_ → Si–O–Si + H_2_O (9)

where R and R′ represent alkyl groups.

Silica gel and microspheres have been prepared successfully from TEOS through non-aqueous sol gel process using formic and acetic acids as the reactants, solvents, and catalysts [206,207]. Some water is generated during the reaction as by product and it has been shown that this in situ water made the sol to gel transformation relatively faster than the aqueous sol-gel process. The nature of carboxylic acid changes the gelation time in the following order [207]: Propanoic acid > acetic acid > formic acid. Biocompatible and degradable, yet water-insoluble polymers can be covalently crosslinked through a non-aqueous sol-gel process (Figure 3D) [78]. The amount of water generated in this process is very small and should not cause any phase separation. The amount of acid needs to be optimized carefully thus that the resultant O/I class II hybrids do not pose any cytotoxic effect.

##### Incorporation of Necessary Components

**Figure 5:**
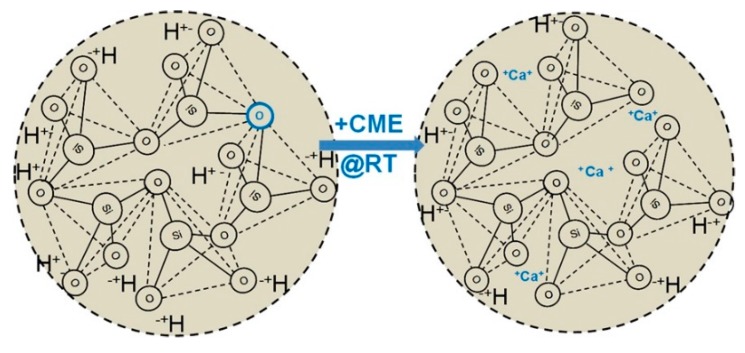
Schematic illustration of calcium incorporated in the glass network at room temperature using calcium methoxyethoxide (CME) as a calcium source in the sol-gel synthesis [209]. Reproduced with permission from the cited reference.

Most of O/I class II hybrids reported in the literature are polymer/SiO_2_ based hybrids. Synthesis of polymer/BG class II hybrids is challenging due to lack of appropriate calcium precursor for the sol-gel process [59]. Calcium nitrate tetrahydrate (Ca(NO_3_)_2_·4H_2_O) has long been used as a calcium precursor to synthesize BG through the sol-gel process. However, it requires to be heated above 400 °C to incorporate calcium as a part of the inorganic BG network [131,168,208,209], which is not possible for O/I hybrids because such a high temperature will decompose the polymer. Calcium chloride was also utilized as a calcium source to prepare class II hybrids [172] but calcium was not bonded with O/I network due to the low temperature of sol-gel process. Organic salts of calcium, such as calcium methoxyethoxide have been used to synthesize hybrids (Figure 5), but its high reactivity to water and hence instability was a major challenge for not utilizing it during the sol-gel process [210,211]. Calcium ethoxide is a calcium alkoxide that has been investigated as a calcium source. Although calcium ethoxide is also highly reactive towards water, recent studies have tried to control the reaction rate to some extent by working in dilute conditions and providing minimal amount of water, to be able to provide time for processing of the hybrid prior to complete gelation [169,212]. Calcium does not necessarily need to be chemically bonded with the O/I network to make the hybrid bioactive and osteogenic, but the dissolution rate of calcium should be congruent with other bioactive components from O/I hybrid biomaterial scaffolds. This can be achieved by higher degree of crosslinking between organic and inorganic phases thus that calcium will be entrapped within the backbone. A lower amount of calcium has also been incorporated in a hybrid of γ-PGA and silica through chelation by using calcium salt of γ-PGA in the synthesis process [213].

To compensate for the absence of calcium due to the challenges of its incorporation in the hybrid, other elements have been investigated to be incorporated in the glass network. For instance, boron-containing BG has excellent bioactivity and promotes cell attachment [146,214]. Boron can be incorporated in O/I hybrid biomaterial through the sol-gel process (Figure 2B,D) [215,216]. Organic precursors of boron, such as trimethyl borate or triethyl borate can be introduced in the sol-gel process and chemically linked to O/I hybrid matrix as network modifier. Strontium doped BG also has shown significant upregulation of bone-related gene expression when seeded with mouse osteoblast [217]. Strontium isopropoxide as a precursor of strontium can also be introduced into the hybrids through the sol-gel process. 

## 4. New Materials Are Emerging for Bone Tissue Engineering Scaffolds

### 4.1. Mesoporous Materials

Although mesoporous materials are not emerging materials per se, their utility in bone tissue engineering is a newer addition to the plethora of biomaterials. The use of inorganic phases with mesoporosity (pore size in the range of 2–50 nm), such as mesoporous bioactive glass (MBG) is an approach for enhancing the desirable properties of composites such as bioactivity or improvement of mechanical properties. In addition, mesoporous materials have a high surface area due to their porous structures and this feature has been exploited for drug loading application. Therefore, incorporation of mesoporous materials can provide the possibility of drug loading into the O/I composite scaffolds [218,219]. In order to synthesize MBG, a surfactant is introduced in the sol-gel process of the glass as the structure directing agent. After removal of the surfactant at the end of the process by calcination or extraction, mesopores would be created in the places occupied previously by the micelles of the surfactant. MBG can be incorporated in a polymeric matrix in the form of particles [220] or can be coated on the surface of the polymeric scaffold [221], or can be fabricated as a scaffold itself and then be coated with a polymer [222].

### 4.2. Piezoelectric Materials

The piezoelectric effect of bone was first observed in 1957 and attributed to the piezoelectric property of collagen [223]. However, the use of piezoelectric materials for biomedical applications and specifically for bone tissue engineering is a field of research in its infancy. Piezoelectric materials exhibit electrical signals upon application of mechanical pressure on them (direct piezoelectric effect) and mechanical signals upon administration of electrical signals (converse piezoelectric effect). Different piezoelectric materials have been used for bone tissue engineering thus far. Piezoelectric synthetic polymers include PVDF (poly(vinylidene fluoride)), P(VDF-TrFE) a copolymer of vinylidene fluoride (VDF) and trifluoroethylene (TrFE), PHBV (poly-3-hydroxybutyrate-3-hydroxy valerate), Poly-l-lactic acid (PLLA), and piezoelectric natural polymers include cellulose, collagen, silk, and chitin. Piezoelectric ceramics include barium titanate (BT), hydroxyapatite (HA), zinc oxide (ZnO), lead zirconate titanate (PZT), boron nitride nanotubes (BNNT), potassium sodium niobate (KNN), and lithium sodium potassium niobate (LNPN). Lead-containing ceramics, although offering high piezoelectric constant are not useful for tissue engineering applications due to their high toxicity. The common concern in the use of piezoelectric materials for tissue engineering is the biocompatibility, as well as their degradability, since many of these materials are not degradable and for degradable natural polymers, the source of material and proper processing to avoid immunogenic response is required. Moreover, the processing of the material to form a 3D porous scaffold is another challenge. Similar to the piezoelectric effect, the magnetoelectric effect is another emerging area in the field of smart materials for biomedical applications, in which the electrical polarization and magnetization are coupled together. Piezoelectric and magnetoelectric materials have been reviewed recently [224,225,226] and interested readers are directed to them.

### 4.3. Conductive Materials

Conductive materials based on graphene and its derivatives such as graphene oxide (GO), reduced graphene oxide (rGO), carboxyl graphene (CXYG), and carbon nanotubes and quantum dots have been investigated extensively for tissue engineering application. Graphene is a layered material of carbon atoms in a honeycomb structure and with sp^2^ hybridization. Apart from conductivity, these materials possess excellent thermal, mechanical, and optical properties. Initially, they have been incorporated in the composite systems to mainly enhance mechanical properties. However, in recent years, many studies have investigated exploiting their conductivity for enhancement of tissue regeneration. Although many studies have focused on the cytocompatibility of these materials, still conclusive results on long-term in vivo effects of them cannot be found. In addition, due to their non-degradability, their ultimate fate in vivo is not clear. However, it can be concluded that size, shape, and concentration are the determining factors in the extent of toxicity or cytocompatibility of these materials. Oxidation of graphene (GO), reduction (rGO), and introduction of carboxyl functional groups (CXYG) increase graphene hydrophilicity. 

**Figure 6:**
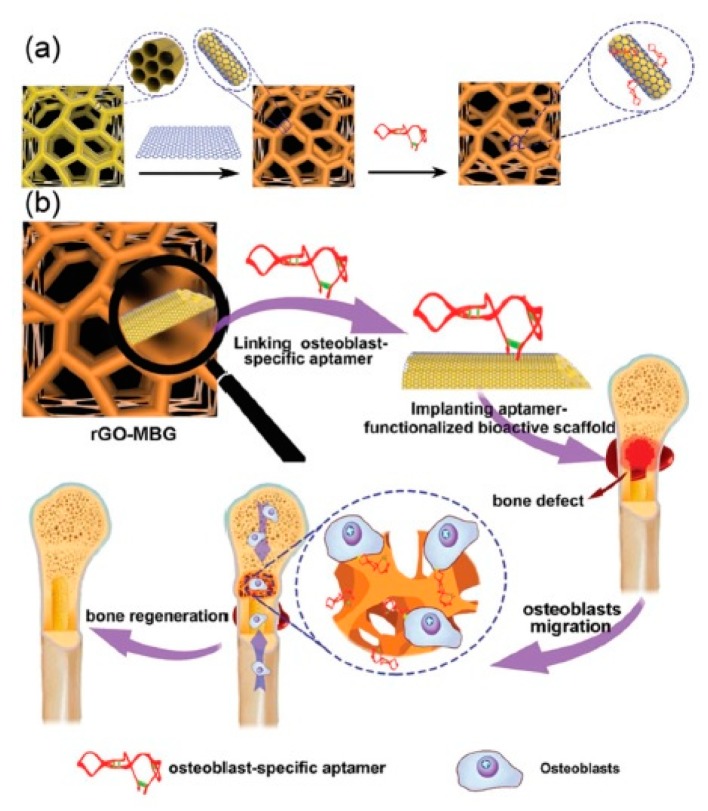
Schematic illustration of mesoporous bioactive glass (MBG) scaffold coated with reduced graphene oxide (rGO) and conjugated with osteoblast-specific aptamer [228]. Reproduced with permission from the cited reference.

Conjugated polymers are another class of conductive materials. Polypyrrole (PPy), polyaniline (PANI) and polythiophene and their derivatives are the most investigated conducting polymers for biomedical applications. These polymers mostly are accompanied by other nonconductive polymers to enhance their degradability, processability, and mechanical properties. Biomaterials with conductive components are particularly interesting for the regeneration of electro-responsive tissues such as cardiac and skeletal muscles, skin, nerves, and bone. For instance, osteogenic precursor cells (MC3T3-E1) cultured on poly(3,4-ethylenedioxythiophene) polystyrene sulfonate (PEDOT:PSS) scaffolds fabricated by freeze-drying, showed enhanced gene expression of osteogenic markers ALPL, Col1A1, and Runx2 alongside increased mineralization and eventually differentiation into mature osteoblasts [227]. Scaffolds of MBG fabricated by use of a sacrificial polyurethane foam were coated with rGO and conjugated with an osteoblast-specific aptamer CH6. These scaffolds result in upregulation of osteoblast markers Col1A1, ALP, Runx2, and Bgla (bone gamma-carboxyglutamate protein) and the rGO coating increased the compressive Young’s modulus form ~2–80 kPa (Figure 6) [228].

Conductive materials, their chemistry, and their application in biomedical fields have been reviewed extensively recently and the interested readers are encouraged to check these references [229,230,231,232]. 

## 5. Successful Bone Repair and Regeneration Requires Appropriate Primary and Stem Cell Sources

For the ultimate goal of bone repair and regeneration, in addition to the scaffold, bone-forming cells are required to form new tissue and remodel the biomaterial. In the case of bone formation and remodeling osteoblast and osteocyte are the main stakeholders to produce bone ECM. Therefore, osteoblasts and/or their precursors are the primary cell sources to engineer bone in vitro and in vivo [233]. Stem cell candidates as osteoblast precursors for bone tissue engineering include bone marrow-derived stem cells (BMSC), adipose-derived stem cells (ASCs), cortical bone fragments mesenchymal stem cells (CBF-MSCs) [234,235], induced pluripotent stem cells (iPSCs), and embryonic stem cells (ESCs). The use of stem cells from different sources for tissue regeneration is an active research topic.

BMSC can differentiate into multiple mesenchymal tissue lineages, including primary osteoblasts and form bone at physiologic condition [236]. Its ease of accessibility and assurance of bone-forming ability made them the most common choice for engineering bone [237]. BMSCs are usually harvested from the marrow aspirate and grow on tissue culture plates and can reach up to 50 population doublings in culture [238]. The initial number of BMSCs collected from bone marrow and their differentiation potency to osteoblast lineage reportedly decline with the patient age [239], which pose fundamental challenges on their use in a clinical setting [240]. It is necessary to apply the suitable cell phenotype for engineering bone tissues, but the exact phenotypic features are not always well-defined. For regeneration of bone, the desired features include good biosynthetic activity (for further development and integration into the scaffolds), expression of osteogenic markers (required for the development of bone ECM), and phenotypic steadiness (to avoid non-specific tissue development) [241].

As an alternative to BMSCs, ASCs are an easily accessible and abundant source of autologous osteogenic cells [242,243] with the same osteogenic differentiation capability as BMSCs [244]. ASCs may survive in low oxygen and/or glucose environments which are advantageous to fabricate bone constructs in vitro [245]. However, ASCs and BMSCs have known limitations, for instance, their proliferation and differentiation potentials are limited and decrease with passages, restricting their application in regenerative medicine [246]. This could be addressed with ESCs; however, ESCs cannot be established from adult cells, therefore it is impossible to make patient-derived ESCs to be used. Moreover, there are ethical and legal constraints on the use of ESCs for tissue engineering. Teratoma formation due to the uncontrolled proliferation of ESCs is another obstacle for the use of them safely in tissue engineering. Induced pluripotent stem cells (iPSCs) generated from adult somatic cells (e.g., human dermal fibroblasts) even after their terminal differentiation have unlimited proliferation and differentiation potential equivalent to ESCs [247,248,249]. However, the use of both ESCs and iPSCs for tissue regeneration purposes is in its infancy and safety issues such as the potential tumorigenesis and efficient differentiation through well-defined and standardized protocols should be addressed before their translation to the clinic. Differentiation of these stem cells prior to transplantation of the engineered tissue is an approach suggested for eliminating the possibility of teratoma formation. However, these terminally-differentiated cells might increase the possibility of immune rejection [250]. Several recent publications have reviewed the use of iPSCs for bone tissue engineering and the interested readers are encouraged to check them [251,252,253].

Another cell source for bone tissue engineering is trabecular bone-derived progenitor cells (TBPCs). Progenitor cells isolated from the trabecular bone have osteogenic potential that can be used to engineer new bone. These TBPCs displayed higher osteogenic potential with ALP expression than BMSCs in vivo and more ectopic bone was formed after five weeks of transplantation [254]. Despite its availability and easy processing, harvesting TBPCs also causes donor-site morbidity similar to BMSCs.

## 6. Future Directions

The field of organic/inorganic hybrids has made considerable progress over the last years. The sol-gel process, which was initially limited to some water-soluble polymers such as PVA has now expanded to several natural and synthetic polymers either water-soluble or insoluble. Many different inventions have been made in the synthesis procedure to modify the properties of the final hybrid material. However, most of the studies focused on the characterization of material for the final application of bone tissue engineering and only a few studies have been devoted to the detailed and systematic investigation of the chemistry of the sol-gel process, the extent of different concurrent reactions and the utilization of advanced chemical analytical techniques for better understanding of the process. These kinds of studies would pave the way for the introduction of complex organic components to the O/I hybrids. On the other hand, scaffold fabrication is still one of the main obstacles on the way towards employing hybrid materials in bone tissue engineering. Additive manufacturing techniques are promising approaches for scaffold fabrication; however, in the case of hybrid material most studies have only used indirect 3D printing due to different requirements for a material to be used as an ink for 3D printing. In addition, since most of the studies have focused on the bioactivity, biocompatibility, and mechanical properties of the hybrid, future works should also focus on detailed and long-term biological response of the cells to the hybrid material. 

Piezoelectric and conductive materials are new “smart materials” and their potential in the biomedical application is being explored. However, still many aspects of them have not been studied. For instance, in the case of conductive materials for bone tissue engineering, only a few studies have actually employed the conductivity of the material for investigating the effect of external electrical stimuli on the cells and even those studies are limited to film-like structures of conductive material and similar studies for 3D scaffolds are missing [255,256,257].
